# Is inadequate play area in schools associated with overweight among students in Addis Ababa, Ethiopia? A comparative cross-sectional study

**DOI:** 10.4178/epih.e2018017

**Published:** 2018-05-12

**Authors:** Tsedey Moges, Bereket Gebremichael, Solomon Shiferaw, Robel Yirgu

**Affiliations:** 1 Public Health Institute, Addis Ababa, Ethiopia; 2 Addis Ababa University College of Health Science, Addis Ababa, Ethiopia

**Keywords:** Adolescent, Overweight, Obesity, School play area, Ethiopia

## Abstract

**OBJECTIVES:**

The prevalence of childhood obesity has more than doubled since it was formally recognized as a global epidemic in 1997. With the increasingly dwindling space for private schools in Ethiopia, unresolved concerns exist among the public regarding the possible effect of limited play areas in schools on overweight/obesity. This study intended to determine and compare the levels of overweight/obesity among adolescents in private schools with and without adequate play area in Addis Ababa, Ethiopia.

**METHODS:**

A school-based comparative cross-sectional study was conducted among 1,276 adolescents. Twenty private schools were grouped into 2 groups based on the size of the play area. Data were collected using a pre-tested questionnaire and anthropometric measurements and analyzed using descriptive statistical tests and logistic regression.

**RESULTS:**

The magnitude of overweight/obesity was significantly higher in schools with inadequate play area (19.4%; 95% confidence interval [CI], 16.4 to 22.7) than in schools with adequate play area (14.6%; 95% CI, 11.9 to17.5). Inadequacy of the play area was also positively associated with overweight/obesity in the multiple logistic regression analysis (odds ratio [OR], 1.62; 95% CI, 1.05 to 2.51). Using private car transportation to and from school (OR, 2.27; 95% CI, 1.13 to 4.57), father’s educational status (secondary school and above: OR, 2.54; 95% CI, 1.14 to 5.62), and middle wealth quintile (OR, 2.54; 95% CI, 1.50 to 4.33) were other factors significantly associated with overweight/obesity.

**CONCLUSIONS:**

Inadequate play area in schools was an important contributor to overweight/obesity. Sedentary behavior was also significantly associated with overweight/obesity.

## INTRODUCTION

The World Health Organization (WHO) recognized obesity as a global epidemic in 1997 [1]. In the past 30 years, the prevalence of obesity has doubled in children and quadrupled among adolescents [2]. Currently, nearly 30% of the world population is estimated to be either overweight or obese, of which two-thirds are living in developing countries [3].

In Ethiopia, especially in Addis Ababa, as a result of urbanization, priority is shifting to using space for new buildings, condominiums (which represent a dramatic shift in housing), roads, and car parks instead of children’s recreational and play areas [4]. Similarly, with increasingly dwindling space for private schools in Ethiopia, unresolved concerns exist among the public regarding the possible effect of limited play areas in schools on children’s sedentary behavior and its consequences, including overweight/obesity.

Previous studies reported a prevalence of obesity ranging from 4 to 23% among in-school adolescents in Ethiopia, with a higher prevalence observed in private schools (10-23%) [5-8]. In-country studies have measured the magnitude of this phenomenon and its individual-level determinants, but the association between the adequacy of play area in schools and overweight/obesity has not been studied. Nonetheless, sustainable prevention of overweight/obesity results from interventions targeting both individual-level and environmental factors [9].

Therefore, the present study intended to determine and compare the levels of overweight/obesity among adolescents in private schools with and without adequate play area in Addis Ababa. The school environment clearly affects the level of physical activity and overweight/obesity of in-school adolescents, since they spend the majority of their time in schools, making it necessary to explore this association [10].

## MATERIALS AND METHODS

### Study area and period

The study was conducted in private schools in Addis Ababa from February to March 2016.

Addis Ababa is the capital and the largest city of Ethiopia, with an area of 530.14 km^2^. The city has 3 administrative layers: 1 city government, 10 sub-cities, and 116 *woreda* administrations. The 2017 population projection of Ethiopia estimated Addis Ababa to have a total population of 3,434,000 urban inhabitants, including 1,625,000 males and 1,809,000 females [11].

In 2016 there were 2,154 schools, of which 462 were governmental, 28 public, and 1,664 privately owned in Addis Ababa. Of the private schools, 930 were kindergartens, 591 were primary-level (grades 1-8), 143 were secondary-level (grades 9-12), and 57 had both primary- and secondary-level education programs. Government schools refers to those that are totally under the government control regarding its budget and administration whereas public school is a no-fee school, funded both the government, non-government bodies but operated by the state.

### Study design

We conducted a school-based comparative cross-sectional study that involved 2 groups of private schools categorized by the adequacy of the play areas on their grounds.

### Population

All private school adolescents aged 10-19 years during the study period were the source population for this study. The study population was all adolescents aged 10-19 years in selected private schools in Addis Ababa during the study period.

Adolescents with less than 1 year of attendance in the selected schools and those with a visible deformity were excluded.

### Sample size calculation

The sample size was calculated using a double-population proportion formula with the following assumptions: P_1_=50% (proportion of overweight/obese adolescents in schools without ade2quate play area), P_2_=40% (proportion of overweight/obese adolescents in schools with adequate play area). These assumptions were made because of the lack of similar studies in similar study areas. Taking a pooled population proportion of P=45%, Z α/2=1.96 at a 95% level of confidence, Z_β_=0.84 for 80% power, and r=1 (the proportion of adolescents in schools with and without an adequate play area was taken as equal, or 1:1), n_1_ was calculated to be 408 and n_2_=408, resulting in a total n=816. Adding a 5% non-response rate gave a total sample size of 857, and considering a design effect of 1.5, the final sample size was calculated to be 1,286.

### Sampling technique

A 3-stage random sampling technique was used to select study participants. In Addis Ababa, there were 57 private schools that had both primary- and secondary-level education programs.

We first classified these schools into 2 strata: those with adequate play area and those without adequate play area, according to the Addis Ababa Education Office primary and secondary school play area (space) requirement. Accordingly, schools with a volleyball, basketball, and handball play area or a total schoolyard of 1,092 m^2^ were classified as having adequate play area and those with a schoolyard of less than 1,092 m^2^ were classified as having inadequate play area [12]. According to these criteria, 33 of the schools had adequate play area, while 24 did not. We randomly selected a total of 20 schools: 10 with adequate play area and 10 without adequate play area.

The total sample size was equally allocated between schools with and without adequate play area (643 for each category). Then, the sample size was allocated for each grade proportionally to the sum of students in each grade. We selected 1 section from each grade randomly, by the lottery method. Finally, we selected study participants by simple random sampling, using the lottery method, from the selected sections within each grade. The students’ class list was used as a sampling frame for random selection.

### Data collection and quality assurance process

A pretested structured questionnaire was used to measure socio-demographic and socioeconomic characteristics. Data on household fixed assets and housing conditions were collected to estimate the wealth of each household.

The WHO Global Physical Activity Questionnaire (GPAQ) was adapted to assess the physical activity and sedentary behavior of the adolescents.

Anthropometric measurements were made using calibrated digital weight balances and measuring sticks. Body weight measurements were made with a precision to the nearest 0.1 kg. The measuring scale was calibrated every morning using 2.0 kg iron bars before data collection started. Data collectors also checked whether the scales read 0.0 kg before each measurement. A measuring stick with a precision of 0.1 cm was used to measure height. The students wore light clothes during body weight measurements and height was measured with bare feet.

Procedures were put in place to maintain data quality. Data were collected by 10 data collectors who had completed high school. The principal investigator gave practical training for data collectors regarding the content of the questionnaire, interview techniques, and how to take anthropometric measurements. Standardization of data collection regarding measurement errors was done during the training. The questionnaire was developed in English and translated into the local language (Amharic). It was pretested and changes were made to the original questionnaire based on the findings from the pretest.

Data quality and completeness were assessed in the field on a daily basis during data collection.

### Data analysis and study variables

Data were coded, entered, and cleaned using EpiData version 3.1 (EpiData Association, Odense, Denmark), and all statistical analyses were performed using Stata version 12.1 (StataCorp., College Station, TX, USA).

A descriptive statistical analysis was conducted using frequency, percentage, mean±standard deviation (SD), and median (interquartile range) to describe the study population by explanatory variables and body mass index (BMI) for age Z-score (BAZ) status.

Control variables for socio-demographic characteristics such as age, parent educational status, and family size were analyzed after conversion into categorical variables.

Principal component analysis (PCA) was conducted to transfer the asset information into latent factors, and a wealth score was used for the first PCA explaining most of the variation based on the objectives of the study. Housing conditions, household assets, and services were components of the wealth index considered for the PCA. Participants were divided according to the wealth score into 5 quintiles (lowest, lower, middle, higher, and highest).

Binary logistic regression analysis was carried out using odds ratios (ORs) to assess the associations between dependent and independent variables. Variables with p-values<0.3 in the binary regression analysis were included in the final model of the multiple logistic regression analysis to characterize their independent effects on overweight/obesity after controlling for the observed covariates. Total physical activity per day in minutes and time spent sitting or reclining per day were removed from the final multivariable logistic regression analysis model because they were intervening factors between the adequacy of play area and overweight/obesity, as shown in previous studies.

### Variable definitions

We used the WHO 2007 growth reference values to classify adolescents based on BAZ using WHO AnthroPlus version 1.0.4 (http://who-anthroplus.sharewarejunction.com/). Participants with a BAZ ≤−2 were classified as severely thin, >−2 and ≤−1 as thin, >−1 and <1 as normal weight, ≥1 and <2 as overweight, and ≥2 as obese. The combination of overweight/obesity was the outcome variable for this study, with the remaining categories collectively considered not overweight/obese.

The GPAQ Analysis Guide was used to assess the physical activity level of the study participants. Study participants were asked about the days per week and hours per day they spent on different activities. The activities were grouped under the comprehensive domains of regular physical activity, transportation, leisure time (recreational) activities, and sedentary time spent per day. Minutes spent on activities in the first 3 domains were calculated to give a single variable (total physical activity level per day). Total physical activity was then categorized as high (≥60 min/d), medium (30-59 min/d) and low (<30 min/d) based on WHO recommendations for physical activity in adolescents.

Sedentary time spent per day was also categorized as <8 hours and ≥8 hours (below the mean sedentary time and greater than or equal to the mean sedentary time, respectively).

Means of transportation were analyzed with the variable recoded based on 2-way (from home to school and from school to home) and 1-way (either from home to school or from school to home) transportation. Time spent on television and sleeping time per day were transformed into categorical variables for analysis.

### Ethical considerations

Ethical clearance was obtained from the Addis Ababa University School of Public Health Research Ethics Committee. The purpose of the study was explained to school officials and permission was sought before starting data collection. Informed consent was obtained for adolescents aged 18 and 19 years. For adolescents less than 18 years of age, consent was obtained from their parents, and they were also asked for their assent.

## RESULTS

Out of the 1,286 adolescent students who were selected, 1,277 participated in this study, yielding a response rate of 99.3%. Data from 1,276 participants were analyzed after the data from 1 participant were removed due to incompleteness. The number of participants from schools with adequate play area was 638 (50.0%); the remaining 50.0% of participants were from schools without adequate play area.

### Socio-demographic characteristics

The mean age of the respondents was 14.4±2.4 years, with no significant difference between the adolescents in schools with and without adequate play area. Of the sampled adolescents in schools with adequate play area, the majority (335, 52.5%) were between the ages of 15-19, while the rest (303, 47.5%) were 10-14 years of age. In contrast, in the schools without adequate play area, the majority of the respondents (330, 51.7%) were between the ages of 10-14, while the rest (308, 48.3%) were 15-19 years. There were 293 (45.9%) male and 345 (54.1%) female respondents from schools with adequate play area and 345 (54.1%) male and 293 (45.9%) female respondents from schools without adequate play area. All the respondents were from grades 5-12 (Table 1).

### Physical activity and sedentary behavior

The median total physical activity level per day among the total respondents was 66.1 minutes. Among participants in schools without adequate play area, the median total physical activity level was 57.9 minutes, which was lower than that observed among students in schools with adequate play area (73.4 min/d).

Adolescents were classified by their total physical activity level based on the WHO recommendation of at least 60 minutes of physical activity per day for health. Of the study participants, 54.1% met the WHO recommendations and were classified as having a high level of total physical activity, while the rest were insufficiently active and were classified as having moderate (30-59 minutes) or low (<30 minutes) total physical activity per day. Among the adolescents in schools with adequate play area, 60.5% had a high level of total physical activity per day, which was higher than the corresponding percentage in schools without adequate play area (47.6%). In addition, 15.8% of the adolescents in schools with adequate play area had a low level of daily physical activity, vs. 23.2% of those in schools without adequate play area.

Regarding sedentary time, 56.1% of adolescents in schools without adequate play area spent less than 8 hours per day sitting, which was a higher percentage than was observed in schools with adequate play area (49.7%).

Among the total respondents, 41.8% walked to and from school and 11.1% used a private car as a means of transportation (Table 2).

### Nutritional status of adolescents

The mean±SD of the BAZ of the total participants was −0.21± 1.27.

**Figure 1.**
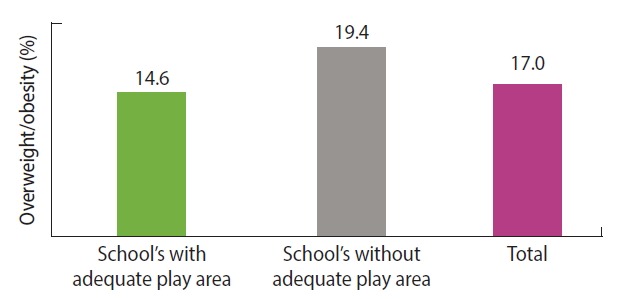
Percentage of overweight/obesity among adolescents in private schools with and without adequate play area in Addis Ababa, Ethiopia.

The overall percentage of overweight/obesity was 17.0% (95% confidence interval [CI], 15.0 to 19.2). It was significantly higher in schools without adequate play area (19.4%; 95% CI, 16.4 to 22.7) than in schools with adequate play area (14.6%; 95% CI, 11.9 to 17.5) (p<0.01) (Figure 1).

**Figure 2.**
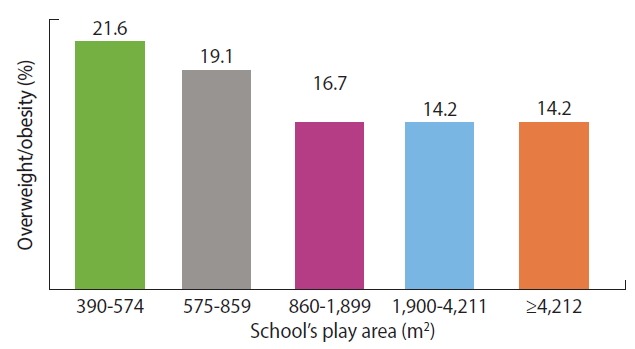
Percentage of overweight/obesity among adolescents compared to measurement of school’s play area in private schools of Addis Ababa, Ethiopia.

Furthermore, schools with larger play areas showed lower levels of overweight/obesity (Figure 2).

### Logistic regression analysis

Binary and multiple logistic regression analyses were performed to identify factors associated with overweight/obesity. Variables with a p-value <0.3 in the binary regression analysis (sex, father’s educational status, socioeconomic status, adequacy of play area, and means of transportation from home to school and from school back to home) were included in the final model for multivariable logistic regression analysis. Total physical activity per day in minutes and time spent sitting or reclining per day were removed from the final model because they were established to be intervening factors between adequacy of the play area and overweight/obesity in previous studies.

In the multiple logistic regression analysis, adolescents who were in schools without adequate play area had 1.6 times higher odds of being overweight/obese than those in schools with adequate play area (adjusted OR [aOR], 1.62; 95% CI, 1.05 to 2.51).

The likelihood of being overweight/obese among those who used private car transportation to and from school was also significantly higher than among those who walked to and from school (aOR, 2.27; 95% CI, 1.13 to 4.57).

Similarly, father’s educational status was positively associated with overweight/obesity. Adolescents whose father’s educational status was secondary school (grades 9-12) or higher (above grade 12) had higher odds of being overweight/obese than those whose fathers had no formal education (aOR, 2.54; 95% CI, 1.14 to 5.62 and aOR, 2.20; 95% CI, 1.10 to 4.40, respectively).

Moreover, adolescents whose parents’ socioeconomic status was within the middle wealth quintile had 2.5 times higher odds of being overweight/obese than those in the lower wealth quintile (aOR, 2.54; 95% CI, 1.50 to 4.33) (Table 3).

## DISCUSSION

Based on the results of this study, inadequate play area in schools was significantly associated with overweight/obesity. Traveling with a private car, higher levels of father’s educational status, and middle socioeconomic status were also factors associated with overweight/obesity.

Overall, our findings are comparable with results from earlier studies. A study conducted among schools in Minnesota in the US found that a lower proportion of neighborhood park/recreation land was associated with higher BAZ. Similarly, a study in Arkansas also found that proximity of neighborhood parks was associated with lower BAZ among rural children [13,14]. In contrary, open space was not associated with BMI in a study conducted in Massachusetts among urban students. However, this may have been because the population density was lower in the area used as an open-space variable, which included a combination of green-space land characteristics, such as forest and parks [15].

Traveling with a private car to and from school was associated with higher odds of being overweight/obese than walking. Comparable to this finding, a community-based study of school children in Brazil also showed those who traveled to school using a car had higher odds of overweight than those who walked/biked to school (aOR, 1.50; 95% CI, 1.14 to 1.91). Other studies in Addis Ababa and Bahir Dar, Ethiopia, similarly reported that using a car as a means of transportation to and from school increased the odds of being overweight/obese by 2-fold (OR, 2.20; 95% CI, 1.40 to 3.40 and aOR, 2.53; 95% CI, 1.26 to 5.06, respectively) [5,16,17].

Higher levels of father’s education (secondary school and above) were positively associated with overweight/obesity. In an international study in 12 countries, a high level of paternal education was associated with 5 times higher odds of being overweight in Kenyan children, while it was protective against overweight in Brazil and the US [18]. This is comparable with the findings of the current study, in that Kenya and Ethiopia are countries with a similar socioeconomic status. Likewise, secondary and higher level educational status of mothers was also associated with higher odds of overweight/obesity in a case-control study in the city of Hawassa, Ethiopia [19]. Parental education is often used as a proxy for socioeconomic status, and in low-income countries, higher socioeconomic levels are usually associated with elevated levels of risk for overweight/obesity.

It was also found that adolescents in the middle wealth quintile had higher odds of overweight/obesity than those in the lower wealth quintiles. A similar result was found in a study conducted in the same area [20]. Another study conducted in Addis Ababa found comparable results, with participants in the middle and higher wealth categories having higher odds of being overweight/obese than those in the lower wealth category. Only higher socioeconomic status was associated with overweight/obesity in a third study in Addis Ababa, which may have been due to the fact that the study was conducted in both private and governmental schools, unlike the current study, which only included private schools [6,7].

As a limitation of this study, temporal relationships could not be identified; in particular, it could not be determined whether participants had recently adopted lifestyle changes after becoming overweight or obese. This consideration could have underestimated the relationship between overweight/obesity and lifestyle factors. A strength of this study is the progress it made in trying to address a growing public health concern in urban Ethiopia by triangulating individual-level and environmental determinants.

In conclusion, the findings of this study yield insights into the effect of inadequate play area in schools as an important contributor to the burden of overweight/obesity. Sedentary behavior was also a significant factor associated with overweight/obesity among private-school adolescents.

We recommend that relevant officials among private school regulatory bodies need to consider having adequate play area as one of the key criteria in licensing and monitoring private schools, along with other academic requirements. Ongoing monitoring should also be conducted of the implementation of existing guidelines regarding play areas in schools. School officials should work on promoting walking to school and an active lifestyle. Further studies assessing other environmental factors that could affect overweight/obesity would also be of great value.

## Figures and Tables

**Table 1.** Socio-demographic characteristics of adolescents in private schools with and without adequate play area in Addis Ababa, Ethiopia

Variable	School had adequate play area		Total (n=1,276)	p-value
	Yes (n=638)	No (n=638)		
Age (yr)				
10-14	303 (47.5)	330 (51.7)	633 (49.6)	
15-19	335 (52.5)	308 (48.3)	643 (50.4)	0.13
Sex				
Male	293 (45.9)	345 (54.1)	638 (50.0)	
Female	345 (54.1)	293 (45.9)	638 (50.0)	0.04
Grade				
5-8	295 (46.2)	349 (54.7)	644 (50.5)	
9-12	343 (53.8)	289 (45.3)	632 (49.5)	0.02
Father’s educational status (n=1,034)				
No formal education	21 (3.9)	40 (8.1)	61 (5.9)	
Primary school (grade 5-8)	23 (4.2)	52 (10.6)	75 (7.2)	
Secondary school (grade 9-12)	122 (22.5)	138 (28.1)	260 (25.1)	<0.01
Above secondary school	377 (69.4)	261 (53.2)	638 (61.7)	
Mother’s educational status (n=1,054)				
No formal education	33 (6.0)	38 (7.5)	71 (6.74	
Primary school (grade 5-8)	42 (7.7)	72 (14.2)	114 (10.8)	
Secondary school (grade 9-12)	150 (27.4)	183 (36.2)	333 (31.6)	<0.01
Above secondary school	323 (58.9)	213 (42.1)	536 (50.8)	
Family size				
≤5	332 (52.0)	329 (51.6)	661 (51.8)	0.87
>5	306 (48.0)	309 (48.4)	615 (48.2)	

Values are presented as number (%).

**Table 2.** Physical activity level and sedentary behavior of adolescents in private schools with and without adequate play area in Addis Ababa, Ethiopia

Variable	School had adequate play area		Total (n=1,276)	p-value
	Yes (n=638)	No (n=638)		
Total physical activity (min/d)				<0.01
High (≥60)	386 (60.5)	304 (47.6)	690 (54.1)	
Moderate (30-59)	151 (23.7)	186 (29.2)	337 (26.4)	
Low (<30)	101 (15.8)	148 (23.2)	249 (19.5)	
Sedentary behavior (time spent sitting or reclining, hr/d)				0.02
<8	358 (56.1)	317 (49.7)	675 (52.9)	
≥8	280 (43.9)	321 (50.3)	601 (47.1)	
Sleeping (hr/d)				0.02
<8	179 (28.0)	136 (21.3)	315 (24.7)	
8-10	426 (66.8)	467 (73.2)	893 (70.0)	
>10	33 (5.2)	35 (5.5)	68 (5.3)	
Time spent on television (hr/d)				0.01
≤2	391 (61.3)	433 (67.9)	824 (64.6)	
>2	247 (38.7)	205 (32.1)	452 (35.4)	
Means of transportation to and from school (n=1,117)				<0.01
Walking	173 (31.2)	294 (52.2)	467 (41.8)	
Public transportation	152 (27.4)	141 (25.0)	293 (26.2)	
Contract taxi/school bus	144 (26.0)	89 (15.8)	233 (20.9)	
Private car	85 (15.3)	39 (6.9)	124 (11.1)	

Values are presented as number (%).

**Table 3.** Factors affecting overweight/obesity among adolescents in private schools with and without adequate play area in Addis Ababa, Ethiopia

Variable	Overweight/obesity		OR (95% CI)	aOR (95% CI)
	Yes	No		
Age (yr)				
10-14	106 (16.8)	527 (83.3)	1.00 (reference)	
15-19	111 (17.3)	532 (82.7)	1.04 (0.66, 1.63)	
Sex				
Male	89 (14.0)	549 (86.1)	1.00 (reference)	1.00 (reference)
Female	128 (20.1)	510 (79.9)	1.55 (1.01, 2.36)^*^	1.47 (0.92, 2.37)
Socioeconomic status (wealth index)				
Lowest	32 (12.5)	225 (87.6)	1.00 (reference)	1.00 (reference)
Lower	38 (14.5)	224 (85.5)	1.19 (0.70, 2.03)	1.18 (0.73, 1.91)
Middle	61 (24.7)	186 (75.3)	2.30 (1.25, 4.26)^*^	2.54 (1.50, 4.33)^*^
Higher	43 (16.9)	212 (83.1)	1.43 (0.94, 2.17)	1.34 (0.82, 2.19)
Highest	43 (16.9)	212 (83.1)	1.43 (0.87, 2.33)	1.42 (0.79, 2.57)
Grade				
5-8	295 (46.2)	349 (54.7)	1.00 (reference)	
9-12	343 (53.8)	289 (45.3)	0.95 (0.64, 1.39)	
Father’s educational status (n=1,034)				
No formal education	21 (3.9)	40 (8.1)	1.00 (reference)	1.00 (reference)
Primary school (grade 5-8)	23 (4.2)	52 (10.6)	1.14 (0.38, 3.41)	1.86 (0.56, 6.15)
Secondary school (grade 9-12)	122 (22.5)	138 (28.1)	1.62 (0.69, 3.77)	2.54 (1.14, 5.62)^*^
Above secondary school	377 (69.4)	261 (53.2)	1.31 (0.67, 2.53)	2.20 (1.10, 4.40)^*^
Mother’s educational status (n=1,054)				
No formal education	33 (6.0)	38 (7.5)	1.00 (reference)	
Primary school (grade 5-8)	42 (7.7)	72 (14.2)	0.76 (0.25, 2.36)	
Secondary school (grade 9-12)	150 (27.4)	183 (36.2)	1.17 (0.47, 2.95)	
Above secondary school	323 (58.9)	213 (42.1)	1.06 (0.50, 2.24)	
Family size				
≤5	332 (52.0)	329 (51.6)	1.00 (reference)	
>5	306 (48.0)	309 (48.4)	1.01 (0.74, 1.37)	
School has adequate play area				
Yes	93 (14.6)	545 (85.4)	1.00 (reference)	1.00 (reference)
No	124 (19.4)	514 (80.6)	1.41 (1.02, 1.97)^*^	1.62 (1.05, 2.51)^*^
Total physical activity (min/d)				
High (≥60)	98 (14.2)	592 (85.8)	1.00 (reference)	
Moderate (30-59)	56 (16.6)	281 (83.4)	1.20 (0.84, 1.73)	
Low (<30)	63 (25.3)	186 (74.7)	2.05 (1.42, 2.96)^*^	
Time spent sitting or reclining (hr/d)				
<8	92 (13.6)	583 (86.4)	1.00 (reference)	
≥8	125 (20.8)	476 (79.2)	1.66 (1.07, 2.59)^*^	
Time spent on television (hr/d)				
≤2	138 (16.8)	686 (83.3)	1.00 (reference)	
>2	79 (17.5)	373 (82.5)	1.05 (0.73, 1.52)	
Sleeping (hr/d)				
<8	58 (18.4)	257 (81.6)	1.00 (reference)	
8-10	149 (16.7)	744 (83.3)	0.89 (0.59, 1.33)	
>10	10 (14.7)	58 (85.3)	0.76 (0.34, 1.73)	
Means of transportation to and from school (n=1,117)				
Walking	67 (14.4)	400 (85.7)	1.00 (reference)	1.00 (reference)
Public transportation	52 (17.8)	241 (82.3)	1.29 (0.73, 2.29)	1.15 (0.70, 1.90)
Contract taxi/school bus	36 (15.5)	197 (84.5)	1.09 (0.72, 1.64)	1.00 (0.63, 1.58)
Private car	31 (25.0)	93 (75.0)	1.99 (1.23, 3.22)^*^	2.27 (1.13, 4.57)^*^

Values are presented as number (%). OR, odds ratio; CI, confidence interval; aOR, adjusted OR.

* p<0.05.
